# Supplementary material for: Sources, propagation and consequences of stochasticity in cellular growth
Source: Nat Commun. 2018 Oct 30;9:4528. doi: 10.1038/s41467-018-06912-9 (PMC6207721; doi:10.1038/s41467-018-06912-9)
Supplement: Supplementary file 1 — Supplementary Information [file 41467_2018_6912_MOESM1_ESM.pdf]

# Supplementary Information:
# Sources, propagation and consequences of stochasticity in cellular growth

Thomas et al.

## Supplementary Note 1: Stochastic modelling of single cell lineages

We lay out a stochastic framework that tracks the biochemical reactions occurring in a single cell over many cycles of growth, DNA replication and cell division. For generality, we consider $R$ reactions involving $N$ molecular species $S_1, ..., S_N$

$$\sum_{i=1}^{N} \nu_{ir}^{-} S_i \xrightarrow{a_r} \sum_{i=1}^{N} \nu_{ir}^{+} S_i, \tag{1}$$

where $r = 1, .., R$ and $\nu_{ir}^{\pm}$ are the stoichiometric coefficients and $a_r$ is the reaction propensity. We describe the stochastic dynamics of intracellular molecule numbers $\mathbf{x}(t) = (x_1(t), x_2(t), \dots, x_N(t))^T$ along a cell lineage by a jump process

$$\mathrm{d}\mathbf{x} = \sum_{r=1}^{R} \boldsymbol{\nu}_r \mathrm{d}Y_r(t) - \mathbf{z}_{D,\mathbf{x},p_D} \mathrm{d}D(t). \tag{2}$$

The first term describes the stochastic dynamics of the intracellular reactions, where $Y_r$ are the number of occurrences of the $r^{th}$ reaction, which follows an inhomogeneous Poisson processes with mean $\int_0^t \mathrm{d}s\, a_r(\mathbf{x}(s))$. The vector $\boldsymbol{\nu}_r = (\nu_{1r}^{+} - \nu_{1r}^{-}, \dots, \nu_{Nr}^{+} - \nu_{Nr}^{-})^T$ yields the change in molecule numbers in the $r^{th}$ reaction. The second term describes the cell divisions and depends on the number of molecules lost in a cell division, $\mathbf{z}_{D,\mathbf{x},p_D}$. The process $D(t)$ counts the number of cell divisions, which will be described below. We will refer to such systems as *reaction-division systems.*

We assume that each molecule is partitioned independently at cell division with probability $p_D$ corresponding to the inherited mass fraction of the daughter cell. The sequence $p_1, p_2, \dots, p_{D(t)}$ consists of independent and identically distributed random variables. We do not distinguish between the two daughters, from which follows that $\Pr(p_D) = \Pr(1 - p_D)$, and hence $E[p_D] = 1/2$ for every cell division $D$. The number of molecules lost in a cell division, which here is denoted by $\mathbf{z}_{1,\mathbf{x},p_1}, \mathbf{z}_{2,\mathbf{x},p_2}, ..., \mathbf{z}_{D(t),\mathbf{x},p_D(t)}$, are independent random vectors following a binomial distribution

$$\Pr(\mathbf{z}_{D,\mathbf{x},p_D} = \mathbf{y}|\mathbf{x}, p_D) = \prod_{i=1}^{N} \binom{y_i}{x_i} (1 - p_D)^{x_i} (p_D)^{x_i - y_i}. \tag{3}$$

To model the number of cell divisions, the counting process $D(t)$, we assume that divisions follow initiation of DNA replication after a fixed period $\tau_{C+D}$. Assuming that all origins are replicated simultaneously with rate $a_{R+1}$, we have

$$D(t) = I(t - \tau_{C+D}) = P\left( \int_0^{t-\tau_{C+D}} \mathrm{d}s\, a_{R+1}(F(s), M(s)) \right), \tag{4}$$

since division follows after each initiation event, where $I(t)$ is the total number of these events and $P(t)$ is a unit rate Poisson process. $F(t)$ denotes the number of fork generations given by $F(t) = F_0 + I(t) - D(t)$, while the number of replication origins is $2^F$. Analogous to Donachie's observation[1], we assume that replication initiation is a tightly controlled process occurring after the concentration of origins ($O = 2^F/M$) is diluted below a critical threshold denoted by $O_c$. We therefore set the propensity of replication initiation to

$$a_{R+1}(F, M) = \alpha H(O_c - 2^F/M), \tag{5}$$

where $H(\cdot)$ is the Heaviside step function. Because we focus on the effects of intrinsic fluctuations of the intracellular reactions, we assume that $\alpha$ is sufficiently large such that initiation of DNA replication follows instantaneously after crossing the threshold.

## Supplementary Note 2: Stochastic analysis of reaction-division systems

In this note we derive a jump diffusion approximation for the intracellular concentrations of reaction-division systems in the form of Langevin equations. We then apply a small noise approximation from which we obtain closed form equations for mean and variances of the process. Finally,

we use this result to determine the time-averaged statistics of concentrations and single-cell growth rates, and provide a noise decomposition that allows us to identify the sources of their variations.

To analyse the dynamics of the intracellular concentrations, we define the total cell mass $M$ and change variables from molecule numbers to mass concentrations via

$$\mathbf{X}(t) = \frac{\mathbf{x}(t)}{M(t)}, \quad M(t) = \sum_{i=1}^{N} m_i x_i, \tag{6}$$

where

$$\mathbf{m} = (m_1, ..., m_N)^T \tag{7}$$

is the vector of molecular masses.

The change of variables allows us to formulate a jump-diffusion approximation of the stochastic process in which reactions change the concentrations of all species continuously while cell divisions interrupt the dynamics by discontinuous jumps. Strictly speaking such an approximation is valid in the limit of large molecules numbers[2], or equivalently in the limit of large mass $M$, and therefore we only consider the leading order terms here. In the following, we consider the contributions of biochemical reactions and cell divisions separately and then combine these effects.

### Contributions of biochemical reactions

First, consider the process (2) in between any two cell divisions ($\mathrm{d}D(t) = 0$) that we will approximate for large $M(t)$. Setting $a_r(\mathbf{x}(t)) = M(t) f_r(\mathbf{X}(t)) + O(M^0)$ we have $\mathrm{d}Y_r(t) \approx M f_r(\mathbf{X}(t))\mathrm{d}t + \sqrt{M f_r(\mathbf{X}(t))}\mathrm{d}W_r(t)$, where $\mathrm{d}W_r(t)$ are independent Gaussian white noise sources. Using this relation in Eq. (2) and applying Ito's formula[3] to Eq. (6), we find that

$$\begin{aligned} \mathrm{d}\mathbf{X} =& (\nu \mathbf{f}(\mathbf{X}) - \lambda(\mathbf{X})\mathbf{X})\mathrm{d}t \\ &+ \frac{1}{M^{1/2}} \sum_{r=1}^{R} \left(\boldsymbol{\nu}_r - \mathbf{X}\mathbf{m}^T \boldsymbol{\nu}_r\right) \sqrt{f_r(\mathbf{X})} \mathrm{d}W_r(t), \\ \mathrm{d}M =& \lambda(\mathbf{X}) M \mathrm{d}t + M^{1/2} \sum_{r=1}^{R} \mathbf{m}^T \boldsymbol{\nu}_r \sqrt{f_r(\mathbf{X})} \mathrm{d}W_r(t), \end{aligned} \tag{8}$$

where we neglected terms of order $O(M^{-1})$ and $\nu_{ir} = (\boldsymbol{\nu}_r)_i$ is the stoichiometric matrix. The drift term in the above equation depends on the instantaneous growth rate $\lambda(\mathbf{X})$ that evaluates to

$$\lambda(\mathbf{X}) = \sum_{i=1}^{N} \sum_{r=1}^{R} m_i \nu_{ir} f_r(\mathbf{X}). \tag{9}$$

It can be verified that Eq. (8) obeys conservation of total-mass fraction $\mathbf{m}^T\mathbf{X} = 1$. Moreover, when all reactions are mass-conserving, $\mathbf{m}^T\boldsymbol{\nu}_r = 0$ for $r = 1, .., R$, there is no cell growth, i.e., $\lambda(\mathbf{X}) = 0$, and we retain the standard chemical Langevin equation[4].

### Contributions of cell divisions

We consider a situation where the concentrations $\mathbf{X}$ change via cell divisions. Denoting the division times by $t_1, .., t_{D(t)}$, the jumps of the concentration process are

$$\begin{aligned} \Delta\mathbf{X}(t_D) =& \mathbf{X}(t_D) - \mathbf{X}(t_D^-), \\ \Delta M(t_D) =& M(t_D) - M(t_D^-), \end{aligned} \tag{10}$$

where we use the shorthand notation

$$\mathbf{X}(t_D^-) = \lim_{\Delta t \to 0} \mathbf{X}(t_D - \Delta t)$$

and assume right-continuous paths. If the fluctuations are small, it is sufficient to focus only the first two jump moments.

The change in mass is given by $\Delta M(t_D) = -\mathbf{m}^T \mathbf{z}_D$, where $\mathbf{z}_D$ represents the vector of molecule numbers lost in a division and is distributed according to Eq. (3). Since $\mathbf{m}^T\mathbf{X}(t_D^-) = 1$ and the expectation values are $E[\mathbf{z}_D|\mathbf{X}(t_D^-), M(t_D^-), p_D] = p_D\mathbf{X}(t_D^-)M(t_D^-)$, $E[p_D] = 1/2$, we find

$$E[\Delta M(t_D)|\mathbf{X}(t_D^-), M(t_D^-)] = -\frac{M(t_D^-)}{2}. \tag{11}$$

The total cell mass is thus halved on average, as expected. For obtaining the second jump moment, we use $\mathrm{Var}[\mathbf{z}_D|\mathbf{X}(t_D^-), M(t_D^-), p_D] = (1-p_D)p_D\mathbf{X}(t_D^-)M(t_D^-)$ and the law of total variance to obtain

$$\begin{aligned} &E[\Delta M^2(t_D)|\mathbf{X}(t_D^-), M(t_D^-)] \\ &\quad= E[\mathrm{Var}[\mathbf{m}^T\mathbf{z}_D|\mathbf{X}(t_D^-), M(t_D^-), p_D]|\mathbf{X}(t_D^-), M] + \mathrm{Var}[E[\mathbf{m}^T\mathbf{z}_D|\mathbf{X}(t_D^-), M(t_D^-)]|\mathbf{X}(t_D^-), M(t_D^-)] \\ &\quad= M(t_D^-)E[(1-p_D)p_D]\left(\sum_{l=1}^N m_l^2 X_l(t_D^-)\right) + M^2(t_D^-)\mathrm{Var}[p] \\ &\quad= M(t_D^-)\frac{1-\mathrm{CV}_p^2}{4}\left(\sum_{l=1}^N m_l^2 X_l(t_D^-)\right) + \frac{1}{4}M^2(t_D^-)\mathrm{CV}_p^2, \end{aligned} \tag{12}$$

which depends explicitly on the intracellular concentrations and the coefficient of variation $\mathrm{CV}_p^2 = \mathrm{Var}[p_D]/E^2[p_D]$ of the inherited mass fraction.

Obtaining the concentration jump moments is more elaborate because their jumps depend on the mass before and after division, $\Delta\mathbf{X}(t_D) = (\mathbf{x} - \mathbf{z})/M(t_D) - \mathbf{x}/M(t_D^-)$. Our strategy is to evaluate $E[\Delta\mathbf{X}(t_D)|\mathbf{X}(t_D^-), M(t_D^-), M(t_D), p] = \mathbf{X}(p_D\frac{M(t_D^-)}{M(t_D)} - 1)$ and then set $M(t_D) = p_D M(t_D^-)$ in the resulting expression, which is correct to leading order in the inverse mass. In effect, the mean concentrations do not change at cell division

$$E[\Delta X_i(t_D)|\mathbf{X}(t_D^-), M(t_D^-)] = 0, \tag{13}$$

for $i = 1, \ldots, N$. A similar calculation gives the second jump moments

$$E[\Delta X_i(t_D)\Delta X_j(t_D)|\mathbf{X}(t_D^-), M(t_D^-)] = E\left[\frac{(1-p_D)}{p_D}\right]\frac{X_i(t_D^-)}{M(t_D^-)}\left(\delta_{ij} - \frac{m_i m_j X_j(t_D^-)}{\sum_l m_l^2 X_l(t_D-)}\right). \tag{14}$$

Note that although molecules are partitioned independently, the concentration process becomes correlated because the total mass fraction couples these quantities. Specifically, the change of mass-fractions satisfies $E[m^T\Delta\mathbf{X}(\Delta\mathbf{X})^T m|\mathbf{X}, M] = 0$ and thus the total mass-fraction, $\mathbf{m}^T\mathbf{X} = 1$, is conserved at cell division. To evaluate the expectation involving $p_D$ we consider small variations about the mean, as it is typically the case for symmetrically dividing cells (see also discussion below). We then obtain $E\left[\frac{(1-p_D)}{p_D}\right] \approx 1 + \mathrm{CV}_p^2$ in terms of $\mathrm{CV}_p$. For simplicitly, we will adopt this limit from here on.

Finally, we define the sequence of random vectors $\boldsymbol{\xi}_1, \ldots, \boldsymbol{\xi}_D$ and random variables $\zeta_1, \ldots, \zeta_D$ with zero mean that denote the mass-independent part of the partitioning errors, as well the zero-mean random variables $\eta_1, \ldots, \eta_D$ that describe variation in the inherited volume fraction. These random variables are mutually uncorrelated and their components satisfy $E[\zeta_{D_1,i}\zeta_{D_2,j}|\mathbf{X}, M] = 0$, and

$$\begin{aligned} E[\xi_{D_1,i}\xi_{D_2,j}|\mathbf{X}, M] &= \delta_{D_1,D_2}\left(1 + \mathrm{CV}_p^2\right)X_i\left(\delta_{ij} - \frac{m_i m_j X_j}{\sum_l m_l^2 X_l}\right), \\ E[\zeta_{D_1}\zeta_{D_2}|\mathbf{X}, M] &= \delta_{D_1,D_2}\frac{1}{4}\left(1 - \mathrm{CV}_p^2\right)\sum_{l=1}^N m_l^2 X_l, \\ E[\eta_{D_1}\eta_{D_2}|\mathbf{X}, M] &= \delta_{D_1,D_2}\mathrm{CV}_p^2, \end{aligned} \tag{15}$$

for any two division indices $D_1$, $D_2$. The contribution of cell divisions can then be written as

$$\begin{aligned} \mathrm{d}\mathbf{X} &= \frac{1}{M^{1/2}}\boldsymbol{\xi}_{D(t)}\mathrm{d}D(t), \\ \mathrm{d}M &= -\frac{M}{2}(1 + \eta_{D(t)})\mathrm{d}D(t) + M^{1/2}\zeta_{D(t)}\mathrm{d}D(t). \end{aligned} \tag{16}$$

Note that in the main text we omit the dependence on $\mathrm{CV}_p^2$ in the first two expressions of Eq. (15), because biologically relevant division errors are smaller than 10%[5–7], such that $\mathrm{CV}_p^2 \ll 1$. Hence this source of variation has negligible effects on intracellular concentrations of symmetrically dividing cells. For lineages of asymmetrically dividing cells, such as budding yeast, the full dependence of Eqs. (12) and (14) has to be taken into account.

### Langevin equations and growth rate

Combining now the contributions from intracellular reactions, Eq. (8), and divisions, Eq. (16), we obtain the following coupled Langevin equations

$$\begin{aligned} \mathrm{d}\mathbf{X} =& (\nu \mathbf{f}(\mathbf{X}) - \lambda(\mathbf{X})\mathbf{X})\mathrm{d}t \\ &+ \frac{1}{M^{1/2}} \sum_{r=1}^{R} \left(\boldsymbol{\nu}_r - \mathbf{X}\mathbf{m}^T\boldsymbol{\nu}_r\right) \sqrt{f_r(\mathbf{X})}\mathrm{d}W_r(t) + \frac{1}{M^{1/2}}\boldsymbol{\xi}_{D(t)}\mathrm{d}D(t), \\ \mathrm{d}M =& \lambda(\mathbf{X})M\mathrm{d}t - \frac{M}{2}(1+\eta_{D(t)})\mathrm{d}D(t) \\ &+ M^{1/2}\sum_{r=1}^{R} \mathbf{m}^T\boldsymbol{\nu}_r\sqrt{f_r(\mathbf{X})}\mathrm{d}W_r(t) + M^{1/2}\zeta_{D(t)}\mathrm{d}D(t), \end{aligned} \tag{17}$$

where $\mathbf{X}$ obeys conservation of total mass-fraction, $\mathbf{m}^T\mathbf{X} = 1$, as explained above. Finally, to obtain a characterisation of the growth rate we apply Ito's formula to $\ln M$. Neglecting terms of order $O(M^{-1})$, we find

$$\mathrm{d}\ln M = \lambda(\mathbf{X})\mathrm{d}t + \frac{1}{M^{1/2}}\sum_{r=1}^{R} m^T\nu_r\sqrt{f_r(\mathbf{X})}\mathrm{d}W_r(t) - \frac{1}{2}(1+\eta_{D(t)})\mathrm{d}D(t) + \frac{1}{M^{1/2}}\zeta_{D(t)}\mathrm{d}D(t). \tag{18}$$

The first line of the above equation is the logarithmic change in the instantaneous mass in between cell divisions. Informally, we write $\Lambda(t) = \frac{\mathrm{d}}{\mathrm{d}t}\ln M$. Using the law of total variance, we can decompose the growth rate variation into the following contributions

$$\mathrm{Var}[\Lambda] = \mathrm{Var}[\lambda(\mathbf{X})] + \sum_{r=1}^{R} \mathbf{m}^T\boldsymbol{\nu}_r E\left[\frac{f_r(\mathbf{X})}{M}\right]\boldsymbol{\nu}_r^T\mathbf{m}. \tag{19}$$

The first term denotes the contribution due to variations in intracellular concentrations, while the second term stems from the reactions contributing to biomass synthesis. For our purpose, the second term can be neglected because of averaging over the large number of these reactions that occur over one division cycle. To see this, assume for instance that these reactions scale as $f_r \sim \gamma$. The first term of the above equation is then of order $\gamma^2$, while the second term is of order $\gamma$ and thus contributes less to the total variation for large $\gamma$.

### Small noise approximation of reaction-division systems

Stochastic differential equations are notoriously difficult to solve analytically. We will therefore employ a small noise approximation[8] which yields closed equations for the means and variances of intracellular concentrations. In the limit of large $M$, we find that Eq. (17) reduces to a set of rate equations

$$\frac{\mathrm{d}\bar{\mathbf{X}}}{\mathrm{d}t} = \nu\mathbf{f}(\bar{\mathbf{X}}) - \lambda(\bar{\mathbf{X}})\bar{\mathbf{X}}, \tag{20}$$

$$\frac{1}{\bar{M}}\frac{\mathrm{d}\bar{M}}{\mathrm{d}t} = \lambda(\bar{\mathbf{X}}) - \frac{1}{2}\mathrm{d}D(t). \tag{21}$$

For finite but large $M$, $\bar{\mathbf{X}}$ equals the mean concentrations and $\bar{M}$ equals the mean cell mass.

Interestingly, we observe that the dynamics of $\bar{\mathbf{X}}$ is independent of $\bar{M}$ and of the division events. Thus the concentrations $\bar{\mathbf{X}}$ reach a steady state given by the solution of

$$\nu\mathbf{f}(\bar{\mathbf{X}}) = \lambda(\bar{\mathbf{X}})\bar{\mathbf{X}}. \tag{22}$$

The mean cell mass $\bar{M}$ increases exponentially between cell divisions

$$\bar{M}(t) = M_0 e^{\lambda(\bar{\mathbf{X}})t}. \tag{23}$$

Since in the deterministic limit, cell divisions must occur after fixed time intervals of $\tau = \ln 2/\lambda$, the constant $M_0$, denoting the mass at cell birth, can be obtained from the delayed effect of initiation

$$M_0 = \frac{e^{\lambda(\bar{\mathbf{X}})\tau_{C+D}}}{2O_c}. \tag{24}$$

This is analogous to Donachie's result[1,9], which states that the mean cell mass increase exponentially with the mean growth rate.

To investigate the stochastic component of the concentration dynamics, we separate the process $\mathbf{X}(t)$ into a deterministic part $\bar{\mathbf{X}}$, and a stochastic component $\boldsymbol{\epsilon}(t)$ as follows

$$\mathbf{X}(t) = \bar{\mathbf{X}} + \frac{1}{\sqrt{M_0}}\boldsymbol{\epsilon}(t). \tag{25}$$

To leading order in $M_0$, the procedure yields the small noise approximation, which reads

$$\frac{\mathrm{d}\boldsymbol{\epsilon}}{\mathrm{d}t} = \mathcal{J}(\bar{\mathbf{X}})\boldsymbol{\epsilon} + \left(\frac{M_0}{\bar{M}(t)}\right)^{1/2} \sum_{r=1}^{R} \left(\boldsymbol{\nu}_r - \bar{\mathbf{X}}\mathbf{m}^T\boldsymbol{\nu}_r\right) \sqrt{f_r(\bar{\mathbf{X}})}\mathrm{d}W_r(t) + \left(\frac{M_0}{\bar{M}(t)}\right)^{1/2} \boldsymbol{\xi}_{D(t)}\mathrm{d}D(t), \tag{26}$$

where $\mathcal{J}(\bar{\mathbf{X}})$ is the Jacobian of the rate equations (20). It now follows that $E[\boldsymbol{\epsilon}(t)] = 0$ and thus $E[\mathbf{X}(t)] = \bar{\mathbf{X}} + O(M_0^{-1})$. From Eq. (26) we can obtain an equation for the covariance $\Sigma = E[(\mathbf{X} - E[\mathbf{X}])(\mathbf{X} - E[\mathbf{X}])^T]$,

$$\frac{\mathrm{d}\Sigma}{\mathrm{d}t} = \mathcal{J}(\bar{\mathbf{X}})\Sigma + \Sigma\mathcal{J}^T(\bar{\mathbf{X}}) + \frac{1}{\bar{M}(t)}\left(\mathcal{D}(\bar{\mathbf{X}}) + \frac{\mathrm{d}D}{\mathrm{d}t}\Gamma(\bar{\mathbf{X}})\right), \tag{27}$$

with the noise matrices for intracellular reactions and cell divisions defined as

$$\mathcal{D}(\bar{\mathbf{X}}) = \sum_{r=1}^{R} \left(\boldsymbol{\nu}_r - \bar{\mathbf{X}}\mathbf{m}^T\boldsymbol{\nu}_r\right) f_r(\bar{\mathbf{X}}) \left(\boldsymbol{\nu}_r - \bar{\mathbf{X}}\mathbf{m}^T\boldsymbol{\nu}_r\right)^T,$$
$$\Gamma_{ij}(\bar{\mathbf{X}}) = \bar{X}_i\left(1 + \mathrm{CV}_p^2\right)\left(\delta_{ij} - \frac{m_i m_j \bar{X}_j}{\sum_l m_l^2 \bar{X}_l}\right), \tag{28}$$

respectively.

#### Estimating lineage-averaged noise statistics

To obtain a representative measure of concentration variation, we employ statistics averaged over the cell lineage. While the mean concentrations are constant, its covariance can be obtained from the time-averaged solution of Eq. (26). To this end, we employ the Laplace transform of the covariance matrix $\Sigma$ defined as

$$\hat{\Sigma}(s) = \int_0^\infty \mathrm{d}t\, e^{-ts}\,\Sigma(t), \tag{29}$$

obtain the time-averaged covariance matrix $\bar{\Sigma}$ using the limit

$$\bar{\Sigma} = \lim_{T\to\infty}\frac{1}{T}\int_0^T \mathrm{d}t\,\Sigma(t) = \lim_{s\to 0} s\hat{\Sigma}(s). \tag{30}$$

Note that since $\Sigma(t)$ is periodic, the second equality corresponds to the average over a division cycle. For brevity, we introduce the linear operator $\mathcal{L}\hat{\Sigma} = \mathcal{J}\hat{\Sigma} + \hat{\Sigma}\mathcal{J}^T$. Using the fact that in the deterministic limit $\frac{\mathrm{d}}{\mathrm{d}t}D(t) = \sum_{n=0}^{\infty}\delta(t - n\tau)$, the Laplace transform of Eq. (26) becomes

$$s\hat{\Sigma}(s) - \Sigma(0) = \mathcal{L}\,\hat{\Sigma}(s) + \left(\int_0^\infty \mathrm{d}t\,\frac{e^{-ts}}{\bar{M}(t)}\right)\mathcal{D} + \left(\sum_{n=0}^{\infty}\frac{1}{\bar{M}(T)}e^{-sn\tau}\right)\Gamma. \tag{31}$$

Solving for $\hat{\Sigma}(s)$ and multiplying by $s$ we find

$$s\hat{\Sigma}(s) = \frac{1}{s-\mathcal{L}}\left[s\Sigma(0) + \left(s\int_0^\infty \mathrm{d}t\, \frac{e^{-ts}}{\bar{M}(t)}\right)\mathcal{D} + \left(s\sum_{n=0}^{\infty} e^{-sn\tau}\right)\frac{\Gamma}{\bar{M}(T)}\right], \tag{32}$$

where $\bar{M}(T) = 2M_0$. By taking the limit $s \to 0$ of the above equation we obtain the time-averaged covariance $\bar{\Sigma}$. In the same limit, the first term in the square brackets vanishes, while the second and third terms are periodic functions, which evaluate to

$$\lim_{s\to 0}\left(s\int_0^\infty \mathrm{d}t\, \frac{e^{-ts}}{\bar{M}(t)}\right) = \lim_{T\to\infty}\frac{1}{T}\int_0^T \mathrm{d}t \frac{1}{\bar{M}(t)} = \frac{1}{2M_0\ln 2}, \tag{33}$$

and

$$\lim_{s\to 0}\left(s\sum_{n=0}^{\infty} e^{-sn\tau}\right) = \frac{1}{\tau}. \tag{34}$$

Combining the last three equations we find that $\bar{\Sigma}$ satisfies a linear set of equations, also known as the Lyapunov matrix equation, which read

$$0 = \mathcal{J}\bar{\Sigma} + \bar{\Sigma}\mathcal{J}^T + \frac{1}{2M_0\ln 2}\left(\lambda\Gamma + \mathcal{D}\right), \tag{35}$$

and determine the time-averaged noise statistics.

### Estimation of growth rate fluctuations

Assuming small fluctuations we can linearise

$$\lambda(\mathbf{X}(t)) = \lambda(\bar{\mathbf{X}} + M_0^{-1/2}\boldsymbol{\epsilon}(t)) = \lambda(\bar{\mathbf{X}}) + \frac{1}{M_0^{1/2}}\sum_{i=1}^{N}\frac{\partial\lambda(\bar{\mathbf{X}})}{\partial\bar{X}_i}\epsilon_i + O(M_0^{-1}). \tag{36}$$

Averaging the above equation over a division cycle, it follows that $E[\lambda(\mathbf{X})] = \lambda(\bar{\mathbf{X}}) + O(M_0^{-1})$ and

$$\mathrm{Var}[\lambda(\mathbf{X})] = \sum_{i,j=1}^{N}\frac{\partial\lambda(\bar{\mathbf{X}})}{\partial\bar{X}_i}\bar{\Sigma}_{ij}\frac{\partial\lambda(\bar{\mathbf{X}})}{\partial\bar{X}_j} + O(M_0^{-2}), \tag{37}$$

where $\bar{\Sigma}$ is obtained from the solution of Eq. (35). Following the same arguments as above, we can neglect the contributions of the mass production to growth rate $\Lambda$ and thus we use only the first term of Eq. (19). We then find

$$\begin{aligned} E[\Lambda] &= \lambda(\bar{\mathbf{X}}), \\ \mathrm{CV}^2[\Lambda] &= \sum_{i,j=1}^{N}\frac{\partial\ln\lambda(\bar{\mathbf{X}})}{\partial\ln\bar{X}_i}\frac{\bar{\Sigma}_{ij}}{\bar{X}_i\bar{X}_j}\frac{\partial\ln\lambda(\bar{\mathbf{X}})}{\partial\ln\bar{X}_j}. \end{aligned} \tag{38}$$

### Identifying the sources of growth variations

To analyse the sources of growth variations, we follow *Komorowski et al.*[10] who investigated the effects of protein degradation using the linear noise approximation of the Chemical Master Equation. Along the same lines, we note that the matrix $\mathcal{D}$, as defined in Eq. (28), can be re-written as a sum over reactions $\mathcal{D} = \sum_{r=1}^{R}\mathcal{D}_r$. We, therefore, decompose the coefficient of variation into additive contributions $\mathrm{CV}^2[\Lambda] = \sum_{r=0}^{R}\mathrm{CV}_r^2[\Lambda]$, each of which satisfy the equations

$$0 = \mathcal{J}\bar{\Sigma}_r + \bar{\Sigma}_r\mathcal{J}^T + \frac{1}{2M_0\ln 2}\mathcal{D}_r, \tag{39}$$

$$\mathrm{CV}_r^2[\Lambda] = \sum_{i,j=1}^{N}\frac{\partial\ln\lambda(\bar{\mathbf{X}})}{\partial\ln\bar{X}_i}\frac{\bar{\Sigma}_{r,ij}}{\bar{X}_i\bar{X}_j}\frac{\partial\ln\lambda(\bar{\mathbf{X}})}{\partial\ln\bar{X}_j}. \tag{40}$$

The matrices $\mathcal{D}_0 = \lambda(\bar{\mathbf{X}})\Gamma$ and $\mathcal{D}_r = \left(\boldsymbol{\nu}_r - \bar{X}\mathbf{m}^T\boldsymbol{\nu}_r\right)f_r(\bar{\mathbf{X}})\left(\boldsymbol{\nu}_r - \bar{\mathbf{X}}\mathbf{m}^T\boldsymbol{\nu}_r\right)^T$ for $r = 1,\ldots,R$ denote the respective noise matrices for cell divisions and biochemical reactions. Note that in contrast to *Komorowski et al.*[10], we explicitly account for effects of cell divisions and dilution.

## Supplementary Note 3: Parameter estimation

We follow the parametrisation of *Weiße et al.*[11] in large parts, which has been obtained from literature values and parameter optimisation. These values describe population averages, but they do not account for the doubling of mass in single cells. Specifically, intracellular association propensities are inversely proportional to cell volume, which we accounted for by scaling the Michaelis-Menten constants in units of mass concentrations. Additionally, we chose the critical origin concentration $O_c$ to closely match absolute protein levels[12]. To further match absolute mRNA levels[12], we scaled the maximum elongation rate $\gamma_{\max}$ from[11] by a factor of 10, which takes into account multiple ribosome-bindings to mRNAs (with ribosome density 1 per 30 codons[13] and assuming 300 codons per mRNA).

We estimated the absolute transcription rates $w_{r,q,e,t}$ and effective resource levels $\zeta$, which scales the scales the thresholds of transcription and translation elongation (see Supplementary Tab. 1), using an adaptive MCMC sampler[14]. Using the SNA we evaluated mean growth rate as a function of nutrient quality $n_s$ describing number of resource molecules produced per nutrient molecule. Inverting this relation allowed us to determine ribosome levels and growth rate fluctuations for a given mean growth rate as well as to estimate the maximum growth rate for high nutrient qualities. We set the objective function as a weighted least square using (i) ribosomal mass fractions measured in Ref. 15 with weights given by the reciprocal of the variance over replicates[15], (ii) coefficients of variation of single-cell growth rates measured in Refs. 16 and 17, and (iii) constraining maximum growth rate to 3.75 doublings per hour. Because error bars for (ii) and (iii) were not available, we assumed standard deviations of 0.025 and 0.1, respectively, for all data points.

The sampled parameter distributions are shown in Supplementary Fig. 1. We used medians of marginal distributions as parameter estimates, which are given in Supplementary Tab. 1. These values were consistent with the modal values of the sampled distributions of pairs of parameters (Supplementary Fig. 1) indicating that all parameters were identifiable. The identifiability of the parameters depends crucially on the constraints imposed by single-cell data, i.e. growth rate variances, which we demonstrate using profile likelihoods[18,19] (Supplementary Fig. 2).

## Supplementary Note 4: Reduced model for ribosome limitation

The reactions of the reduced model are:

$$\varnothing \xrightarrow{\omega_y} m_y, \quad m_y \xrightarrow{d_y} \varnothing, \tag{41}$$

$$p_r + m_y \underset{k_b}{\overset{k_u}{\rightleftharpoons}} c_y, \quad n_y a + c_y \xrightarrow{\varsigma_y} p_r + m_y + p_y, \tag{42}$$

$$\varnothing \xrightarrow{v_m} a, \tag{43}$$

for $y \in \{r, q\}$. The propensities of mRNA degradation, ribosome binding and unbinding are the same as in the full model but only comprise the r- and q-proteins. Transcriptional and translational propensities depend on the resource $a$ as in the main text, but a constant supply of resources with rate $v_m$, Eq. (43), replaces nutrient uptake and metabolism.

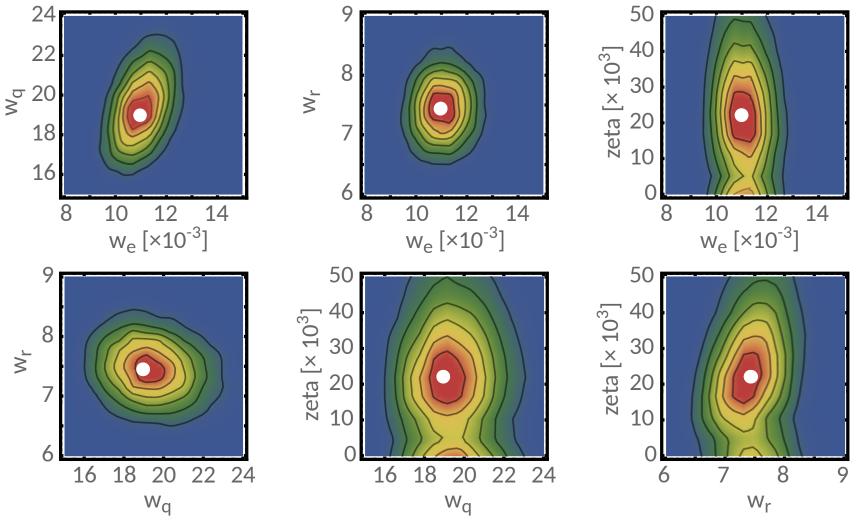


Supplementary Figure 1. **Distributions of sampled parameters.** Smoothed histograms of pairs of parameters obtained from MCMC simulations. Median values of marginal distributions (white filled circles) estimate the parameters, which are in good with modal values of the bivariate parameter histograms.

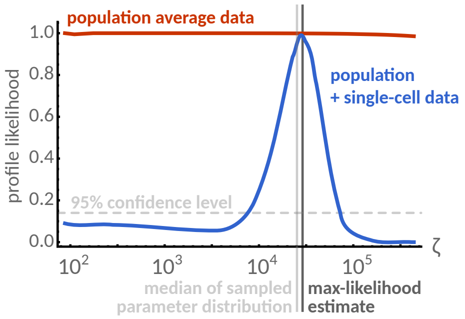


Supplementary Figure 2. **Stochastic model estimates parameters not identified by population averages.** The profile likelihood is given by the maximum likelihood for a fixed value of $\zeta$, a measure of absolute resource levels. Using population average data[15] that constrain only the mean behaviour of the model, the profile is flat (red line) indicating that the parameter is not identifiable. In contrast, including single-cell data[16,17] that also constrain growth rate variances the profile is clearly peaked (blue line) such that the parameter becomes identifiable within a $95\%$ confidence interval (dashed grey line). Also shown is the maximum likelihood estimate (dark grey) and the median of the sampled parameter distributions (light grey line). Likelihood maximisation was performed using the Nelder-Mead method[20].

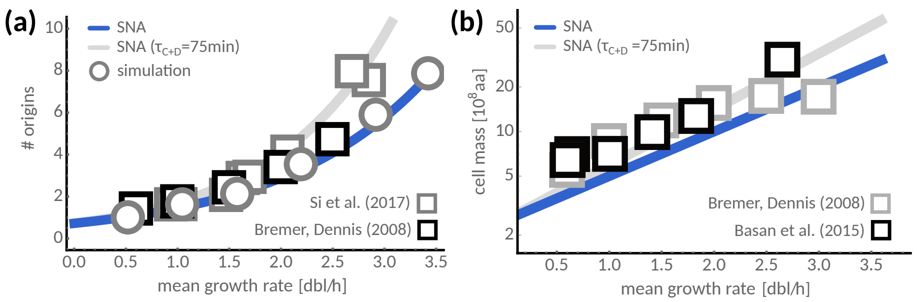


Supplementary Figure 3. **Comparison of predicted DNA content and proteomic mass with experimental data.** **(a)** The mean number of origins at cell birth increases with average growth rate according to $e^{\lambda\tau_{C+D}}/(2\ln 2)$. The theoretical predictions for a $C+D$ period of $60\,\mathrm{min}$ (solid blue line) are in excellent quantitative agreement with stochastic simulations and experimental data in different growth conditions (open black squares[12]). The theoretical prediction (solid grey line) matches recent data from *Si et al.*[21] (open grey squares) when the $C+D$ period is adjusted to the experimentally observed value of $75\,\mathrm{min}$. **(b)** Comparison of predicted birth mass in number of amino acids (aa, lines) proteomics data (open black squares[22], estimates assume an average amino acid weight of $137$Da) and estimates obtained via indirect measurement (open grey squares[12]).

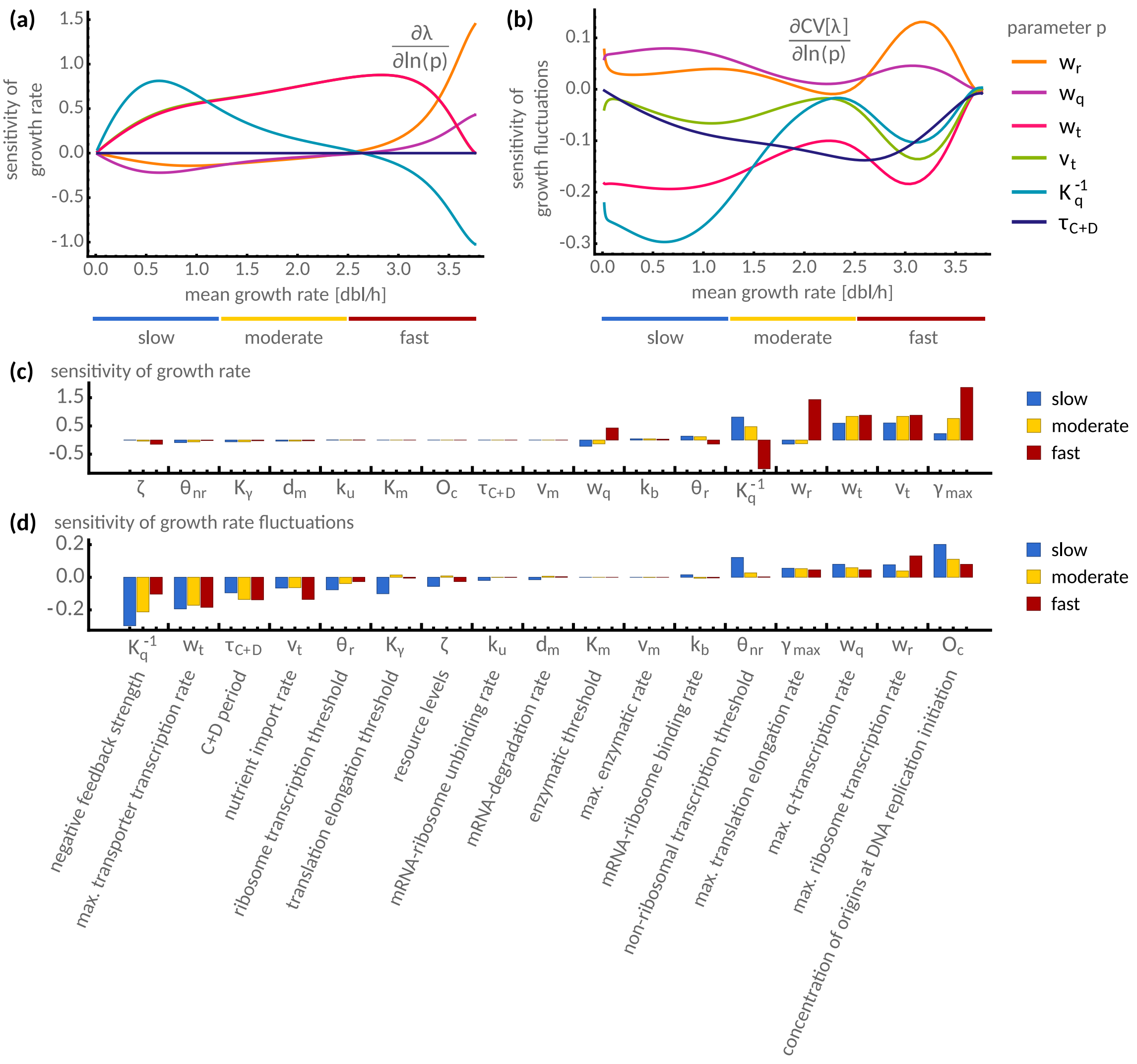


Supplementary Figure 4. **Sensitivities of mean growth rate and fluctuations to variations in model parameters.** **(a)** Sensitivity of the mean growth rate to parameter variations in the transcription rates $w_r$, $w_q$, $w_t (= w_e)$, nutrient import rate $v_t$, negative feedback strength $K_q^{-1}$ and C+D period $\tau_{C+D}$ following DNA replication initiation. **(b)** The corresponding sensitivities of the coefficient of variation of growth rate fluctuations. **(c)** Sensitivity of the mean growth rate $\partial\lambda/\partial \ln p$ to variations in various model parameters (see Supplementary Tab. 1) in various growth conditions. Maximum sensitivity is shown for slow ($0 - 1.25$ dbl/h), moderate ($1.25 - 2.5$ dbl/h) and fast ($2.5 - 3.75$ dbl/h) growth conditions. **(d)** Sensitivity of the growth rate fluctuations $\partial \text{CV}[\lambda]/\partial \ln p$ to variations in all model parameters (cf. Supplementary Tab. 1) in various growth conditions. In all panels sensitivities are computed using finite-difference derivatives of Eqs. (38).

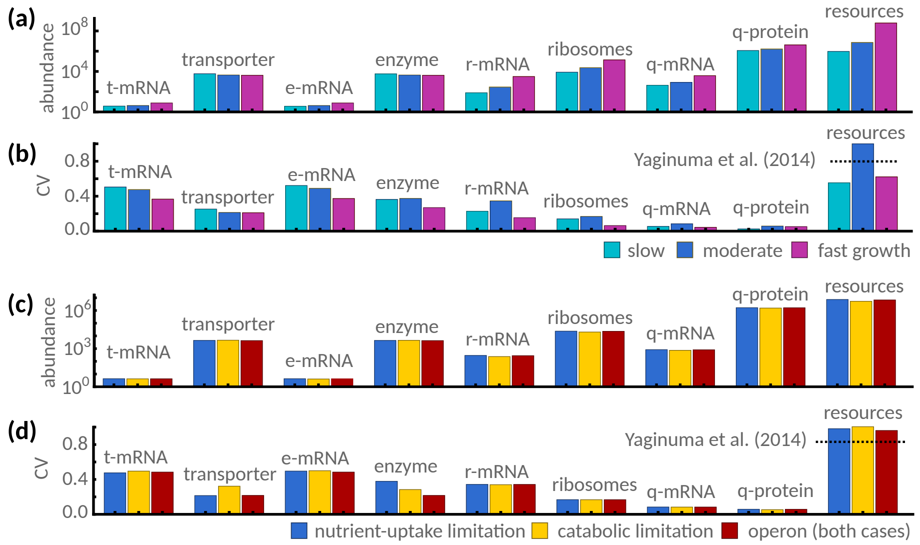


Supplementary Figure 5. **Absolute abundances and concentration fluctuations across growth conditions and under different metabolic limitations.** **(a)** Estimated mean abundances at cell birth range over eight orders of magnitude for three growth conditions (slow $0.7$, moderate $1.4$, fast $3.1$ doublings per hour). Among all groups enzyme and transporter mRNAs were least abundant. Resources are highly abundant, similar to proteins that make up most of the cell mass, such as ribosomes and q-proteins. **(b)** Fluctuations in concentrations of transcriptome, proteome and resources in different growth conditions. The dashed line indicates measured fluctuations in intracellular ATP[23]. **(c-d)** In moderate growth conditions, mean abundances and concentration fluctuations are comparable under limitations of nutrient-uptake (blue bars) and catabolism (yellow). Co-expression of transporter and enzyme mRNA from an operon did not change the results under both limitations (both shown in red).

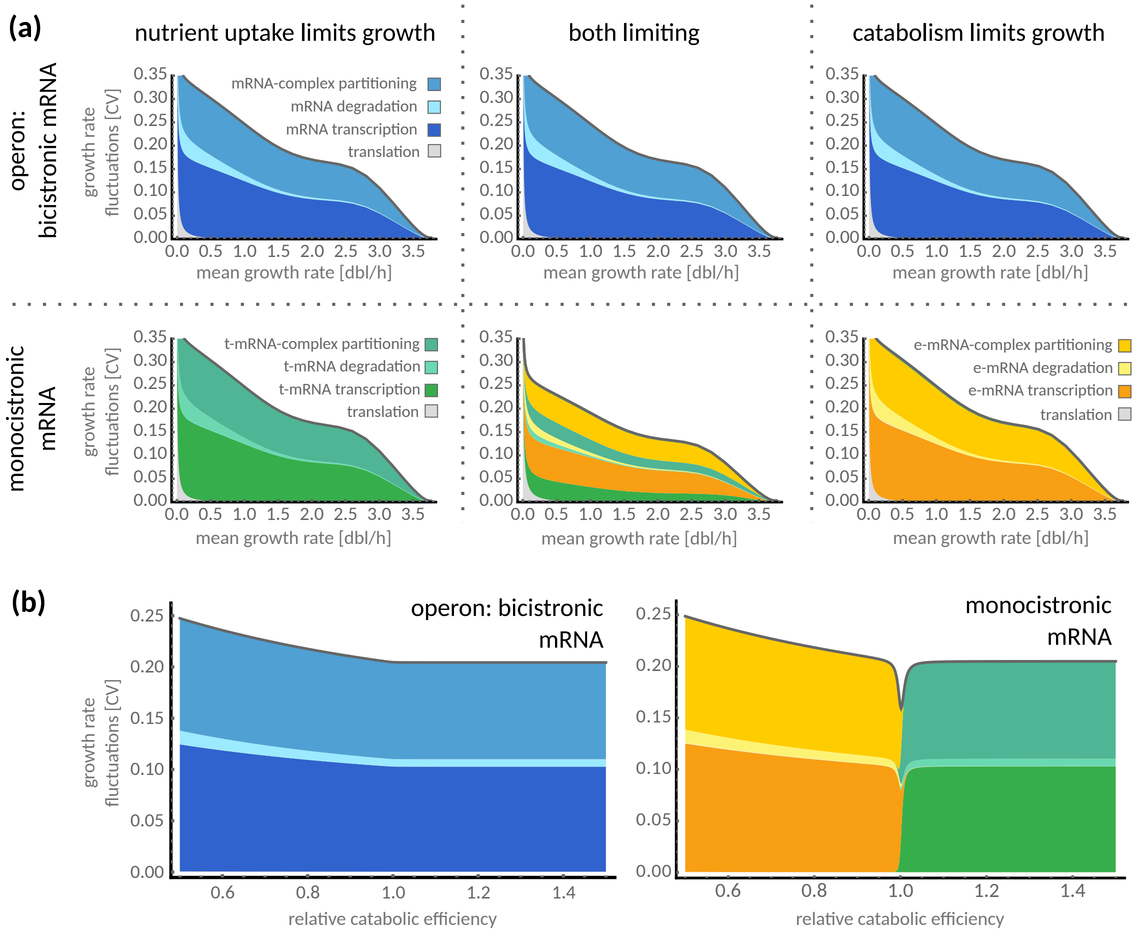


Supplementary Figure 6. **Effect of transporter-enzyme-operon organisation on growth fluctuations.** **(a)** Coefficient of variation (CV) in dependence of the relative rates of catabolic turnover $v_m$ and nutrient uptake rate $v_t$. A detailed breakdown of the sources of growth fluctuations reveals that transcription and translation of co-transcribed bicistronic mRNAs, as well as the stochastic partitioning at cell division of their corresponding mRNA-ribosome complex, are dominating the fluctuations in all conditions ($v_t < v_m$ left, $v_m = v_t$ mid, $v_m < v_t$ right panel). Also the absolute size of fluctuations is comparable. For comparison, we show the dependence for transporters and enzymes being expressed from different promoters (monocistronic mRNAs, lower panel, same as Fig. 3 of the main text). **(b)** Growth fluctuations for enzyme-transporter proteins encoded via monocistronic and bicistronic mRNAs for a representative nutrient condition ($1.5$ doublings per hour). When nutrient uptake is limiting, the size of growth variations is constant (relative catabolic rate $v_m/v_t > 1$) but increases as catabolism becomes growth-limiting ($v_m/v_t < 1$). For monocistronic mRNAs the coefficient of variation exhibits a sharp switch from transporter-limited to enzyme-limited fluctuations (right panel) displaying a noise cancellation effect in the transition region ($v_m/v_t \approx 1$). No such effect is observed for bicistronic mRNAs (left panel).

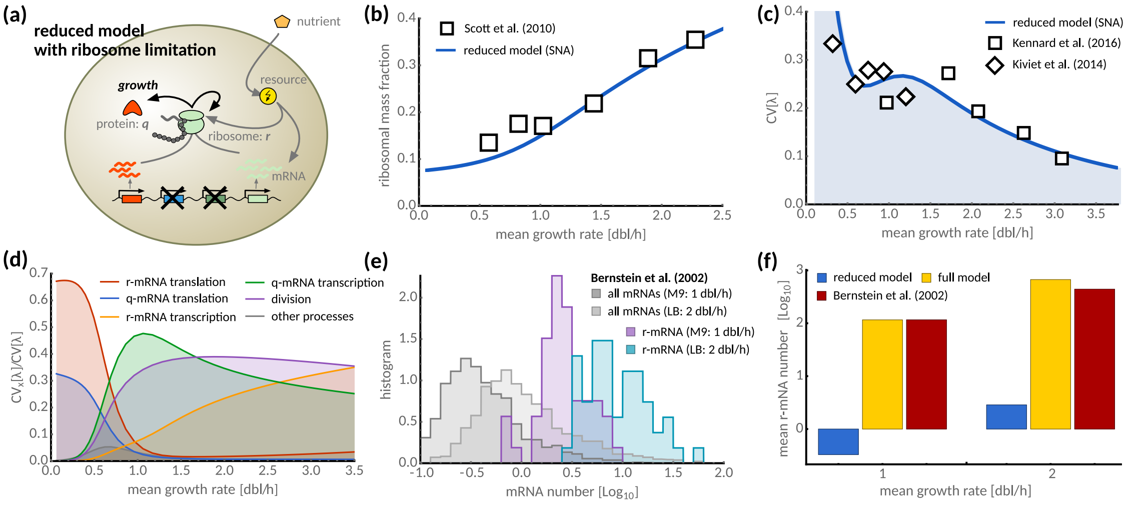


Supplementary Figure 7. **Reduced model with ribosome limitation.** **(a)** Illustration of a reduced model in which transporter and enzyme functions are replaced by a constant inflow of resources. Under these conditions ribosomes are the sole growth-limiting factor. **(b)** The reduced model accurately describes the dependence of ribosome content on mean growth rate (squares, data from[15]). **(c)** The reduced model also recovers growth fluctuations across various conditions (diamonds,[16], squares[17]). **(d)** A detailed breakdown of the sources of growth fluctuations shows that translation of r- and q-mRNAs dominates growth fluctuations in slow growth ($< 1$ dbl/h), while transcription and partitioning dominate in moderate to fast growth conditions ($> 1$ dbl/h). **(e)** Measurements of mean abundances of 54 of the 55 transcripts encoding *E. coli*'s ribosome in two different growth conditions[24]. Ribosomal transcripts (purple for $1$ dbl/h, teal for $2$ dbl/h) are the most abundant mRNAs of the transcriptome (grey). Absolute abundances are obtained by normalising with the total mRNA numbers given in[12] (cf. Fig. 2a of the main text). **(f)** The reduced model (blue) fails to predict the measured total r-mRNA abundances in *E. coli* (red). The r-mRNA levels required to fit (c) and (d) are too small in the reduced model. Therefore, ribosome expression cannot account for the observed growth variation. In contrast, the model presented in the main text is in excellent agreement with the data[24] and supports the assumed nutrient-uptake and catabolic limitations.

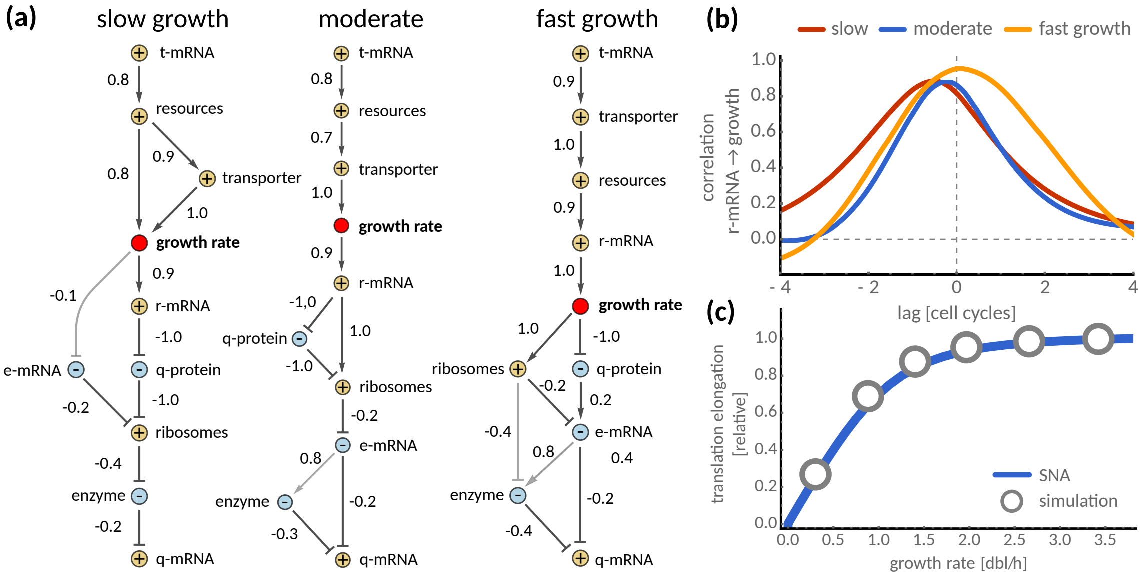


Supplementary Figure 8. **Cross-correlation analysis in different growth conditions. (a)** Minimal delay graph are presented across growth conditions (slow $0.7$, moderate $1.4$, fast $3.1$ doublings per hour). We consistently observe that enzymes, their mRNA and q-proteins are mainly diluted (nodes labelled with $-$ correlate negatively with growth) while all other species increase with growth (nodes labelled with $+$ correlate positively with growth). Further, transporter mRNAs act as a source of fluctuations while q-mRNAs act as a sink due to their negative auto-regulation. **(b)** Moving from slow to fast growth conditions correlations between ribosomal mRNA (r-mRNA) and growth increase and the delay moves from negative to positive values meaning that r-mRNA moves upstream of growth rate. **(c)** This is explained by translational elongation (a function of resource levels) saturating in fast growth conditions. At slow to moderate growth, fluctuations in resources dominate the noise in growth rate, whereas in fast growth conditions noise in ribosomal mRNA concentrations drive growth noise.

| parameter | description | value (n-lim) | value (c-lim) | value (r-lim) | unit | source |
|---|---|---|---|---|---|---|
| $w_r$ | max. ribosome transcription rate | 7.44 | | 74.4 | [1/(aa min)] | ★ |
| $w_q$ | max. $q$-transcription rate | 18.98 | | 189.8 | [1/(aa min)] | ★ |
| $w_t$, $w_e$ | max. enzyme transcription rate | 0.012 | | – | [1/(aa min)] | ★ |
| $\zeta$ | resource levels | 22, 000 | | 440, 000 | [molecs/aa] | ★,▲ |
| $\tau_{C+D}$ | $C + D$ period | 60 | | | [min] | [25] |
| $O_c$ | concentration of origins at DNA replication initiation | 0.2 | | 400 | $[1/(10^8 \mathrm{aa})]$ | |
| $k_{\mathrm{cm}}$ | chloramphenicol-binding rate | 0.0666 | | | $[(\min \mu M)^{-1}]$ | |
| $d_m$ | mRNA-degradation rate | 0.1 | | | $[\min^{-1}]$ | ◇ |
| $m_r$ | ribosome mass | 7459 | | | [aa] | ◇ |
| $m_t$, $m_e$, $m_q$ | mass of non-ribosomal proteins | 300 | | | [aa] | ◇ |
| $v_t$ | nutrient import rate | 660 | | – | $[\min^{-1}]$ | ◇ |
| $v_m$ | max. enzymatic rate | 5800 | 600 | – | $[\min^{-1}]$ | ◇‡ |
| $k_b$ | mRNA-ribosome binding rate | 1 | | | [aa/ min] | ◇ |
| $k_u$ | mRNA-ribosome unbinding rate | 1 | | | $[\min^{-1}]$ | ◇ |
| $K_m$ | enzymatic threshold | 1000 | | – | $[1/(10^8 \mathrm{aa})]$ | ◇ |
| $K_q$ | $q$-autoinhibition threshold | 152 219 | | | $[1/(10^8 \mathrm{aa})]$ | ◇ |
| $\theta_r$ | ribosome transcription threshold | $426.87\zeta$ | | | $[1/(10^8 \mathrm{aa})]$ | † |
| $\theta_{\mathrm{nr}}$ | non-ribosomal transcription threshold | $4.38\zeta$ | | | $[1/(10^8 \mathrm{aa})]$ | † |
| $K_\gamma$ | transl. elongation threshold | $7\zeta$ | | | $[1/(10^8 \mathrm{aa})]$ | † |
| $\gamma_{\max}$ | max. transl. elongation rate | 12600 | | 1260 | [aa/ min] | † |

Supplementary Table I. **Model parameters.** We used default values unless otherwise stated. The 3rd column denotes the parameters value under nutrient-uptake limitation (n-lim), the 4th column denotes the parameters under catabolic limitations (c-lim), and the 5th column denotes values used in a reduced model under ribosome limitation (r-lim, see Supplementary Note 4 and Supplementary Fig. 7). For stochastic simulations with symmetric divisions, we draw the inherited volume fraction from a symmetric Beta distribution with a coefficient of variation of 5%. ▲The parameter $\zeta$ scales the thresholds of transcription, $\theta_r$ and $\theta_{nr}$, and translation elongation $K_\gamma$ and hence is a measure of total resource levels. ★Obtained by parameter optimisation (see Supplementary Note 3). ◇Same as in Ref. 11, †same as in Ref. 11 but rescaled to account for adjusted resource levels; aa unit of amino acid. ‡For mixed nutrient-uptake and catabolic limitations value is rescaled to $600\,\min^{-1}$.

## Supplementary References


[1] W. D. Donachie, "Relationship between cell size and time of initiation of DNA replication," Nature **219**, 1077–1079 (1968).
[2] R. Grima, P. Thomas, and A. V. Straube, "How accurate are the nonlinear chemical Fokker-Planck and chemical Langevin equations?" J Chem Phys **135**, 084103 (2011).
[3] P. Kloeden and E. Platen, *Numerical solution of stochastic differential equations* (Springer, 1999).
[4] D. T. Gillespie, "The chemical Langevin equation," J Chem Phys **113**, 297–306 (2000).
[5] L. Koppes, C. L. Woldringh, and N. Nanninga, "Size variations and correlation of different cell cycle events in slow-growing Escherichia coli." J Bacteriol **134**, 423–433 (1978).
[6] F. J. Trueba, "On the precision and accuracy achieved by Escherichia coli cells at fission about their middle," Arch Microbiol **131**, 55–59 (1982).
[7] J. M. Guberman, A. Fay, J. Dworkin, N. S. Wingreen, and Z. Gitai, "Psicic: noise and asymmetry in bacterial division revealed by computational image analysis at sub-pixel resolution," PLoS Comput Biol **4**, e1000233 (2008).
[8] C. Gardiner, *Stochastic methods* (Springer Berlin, 2009).
[9] S. Taheri-Araghi, "Self-consistent examination of Donachie's constant initiation size at the single-cell level," Front Microbiol **6**, 1349 (2015).
[10] M. Komorowski, J. Miekisz, and M. P. Stumpf, "Decomposing noise in biochemical signaling systems highlights the role of protein degradation," Biophys J **104**, 1783–1793 (2013).
[11] A. Y. Weiße, D. A. Oyarzún, V. Danos, and P. S. Swain, "Mechanistic links between cellular trade-offs, gene expression, and growth," Proc Natl Acad Sci **112**, E1038–E1047 (2015).
[12] H. Bremer and P. P. Dennis, "Modulation of chemical composition and other parameters of the cell at different exponential growth rates," EcoSal Plus **3** (2008), 10.1128/ecosal.5.2.3.
[13] M. Siwiak and P. Zielenkiewicz, "Transimulation-protein biosynthesis web service," PloS one **8**, e73943 (2013).
[14] B. J. Smith and other contributors, *Mamba: Markov chain Monte Carlo for Bayesian analysis in Julia* (2014).
[15] M. Scott, C. W. Gunderson, E. M. Mateescu, Z. Zhang, and T. Hwa, "Interdependence of cell growth and gene expression: origins and consequences," Science **330**, 1099–1102 (2010).
[16] D. J. Kiviet, P. Nghe, N. Walker, S. Boulineau, V. Sunderlikova, and S. J. Tans, "Stochasticity of metabolism and growth at the single-cell level," Nature **514**, 376–379 (2014).
[17] A. S. Kennard, M. Osella, A. Javer, J. Grilli, P. Nghe, S. J. Tans, P. Cicuta, and M. C. Lagomarsino, "Individuality and universality in the growth-division laws of single E. coli cells," Phys Rev E **93**, 012408 (2016).
[18] A. Raue, C. Kreutz, T. Maiwald, J. Bachmann, M. Schilling, U. Klingmüller, and J. Timmer, "Structural and practical identifiability analysis of partially observed dynamical models by exploiting the profile likelihood," Bioinformatics **25**, 1923–1929 (2009).
[19] F. Fröhlich, P. Thomas, A. Kazeroonian, F. J. Theis, R. Grima, and J. Hasenauer, "Inference for stochastic chemical kinetics using moment equations and system size expansion," PLoS Comput Biol **12**, e1005030 (2016).
[20] P. K. Mogensen and other contributors, *Optim: Univariate and multivariate optimization in Julia.* (2014).
[21] F. Si, D. Li, S. E. Cox, J. T. Sauls, O. Azizi, C. Sou, A. B. Schwartz, M. J. Erickstad, Y. Jun, X. Li, *et al.*, "Invariance of initiation mass and predictability of cell size in Escherichia coli," Curr Biol **27**, 1278–1287 (2017).
[22] M. Basan, M. Zhu, X. Dai, M. Warren, D. Sévin, Y.-P. Wang, and T. Hwa, "Inflating bacterial cells by increased protein synthesis," Mol Syst Biol **11**, 836 (2015).
[23] H. Yaginuma, S. Kawai, K. V. Tabata, K. Tomiyama, A. Kakizuka, T. Komatsuzaki, H. Noji, and H. Imamura, "Diversity in ATP concentrations in a single bacterial cell population revealed by quantitative single-cell imaging," Sci Rep **4**, 6522 (2014).
[24] J. A. Bernstein, A. B. Khodursky, P.-H. Lin, S. Lin-Chao, and S. N. Cohen, "Global analysis of mRNA decay and abundance in escherichia coli at single-gene resolution using two-color fluorescent DNA microarrays," Proc Natl Acad Sci **99**, 9697–9702 (2002).
[25] S. Cooper and C. E. Helmstetter, "Chromosome replication and the division cycle of Escherichia coli Br," J Mol Biol **31**, 519–540 (1968).
